# Epidemiology and Antimicrobial Susceptibility of *Salmonella enterica* Bloodstream Isolates Among Febrile Children in a Rural District in Northeastern Tanzania: A Cross-sectional Study

**DOI:** 10.1093/cid/ciy1126

**Published:** 2019-03-07

**Authors:** Omari A Msemo, Joyce Mbwana, Coline Mahende, Anangisye Malabeja, Samwel Gesase, John A Crump, Denise Dekker, John P A Lusingu

**Affiliations:** 1 National Institute for Medical Research, Tanga Center, Tanzania; 2 Centre for International Health, University of Otago, Dunedin, New Zealand; 3 Bernhard Nocht Institute for Tropical Medicine, Hamburg, Germany

**Keywords:** Epidemiology, *Salmonella* Typhi, non-typhoidal *Salmonella*, antimicrobial resistance

## Abstract

**Background:**

*Salmonella enterica* including *Salmonella* Typhi and nontyphoidal *Salmonella* (NTS) are the predominant cause of community-acquired bloodstream infections in sub-Saharan Africa (sSA). Multiple-drug resistance and emerging fluoroquinolone resistance are of concern. Data on the age distribution of typhoid fever in sSA are scarce but essential for typhoid conjugate vaccine policy. We sought to describe *Salmonella* bloodstream infections, antimicrobial resistance, and age distribution at a rural district hospital in northeastern Tanzania.

**Methods:**

From 2008 to 2016, febrile children or children with a history of fever aged 1 month to 5 years admitted to Korogwe District Hospital were enrolled. Demographic, clinical data and blood cultures were collected. Organisms were identified by conventional microbiological methods, and antimicrobial susceptibility test was done by disc diffusion.

**Results:**

Of 4176 participants receiving blood cultures, 383 (9.2 %) yielded pathogens. Of pathogens, 171 (44.6%) were *Salmonella enterica* of which 129 (75.4%) were *Salmonella* Typhi, and 42 (24.6%) were NTS. The median (interquartile range age of participants was 13.1 (6.3–28.0) months for those with *Salmonella* Typhi and 11.5 (8.5–23.4) months for NTS. Of 129 *Salmonella* Typhi, 89 (89.9%) were resistant to amoxicillin, 85 (81.0%) to chloramphenicol, and 93 (92.1%) to trimethoprim-sulfamethoxazole compared with 22 (62.9%), 15 (39.4%), and 27 (79.4%), respectively, for NTS. Multidrug resistance was present in 68 (81.0%) of *Salmonella* Typhi and 12 (41.4%) of NTS.

**Conclusion:**

*Salmonella* Typhi was the leading cause of bloodstream infection among infants and young children <2 years of age admitted to Korogwe District Hospital. Multidrug resistance was common, highlighting a role for typhoid conjugate vaccine into routine infant vaccine schedules.

In sub-Saharan Africa (sSA), *Salmonella* Typhi is a predominant cause of bloodstream infections among both children and adults [1, 2]. Globally, typhoid fever is estimated to cause 17 million illnesses [3] and 178 000 000 deaths annually [4]. *Salmonella* Typhi is transmitted by fecally contaminated water and food. Although there has been progress with improved water and sanitation in sSA since the 1990s [5], many people remain at risk for typhoid fever by consumption of unsafe water and food. Earlier work suggested that risk for typhoid fever in Africa might be concentrated in urban areas and informal settlements [6]. However, recent studies confirm the substantial and underappreciated scale of the problem of rural typhoid fever [7–9]. Although long-term efforts continue to provide microbiologically safe drinking water and food and improved sanitation to those without it, including in difficult to reach rural areas, typhoid conjugate vaccines provide a means to reduce illness and deaths. In October 2017, the World Health Organization (WHO) Strategic Advisory Group of Experts (SAGE) on immunization recommended typhoid conjugate vaccine for routine use in children >6 months of age in typhoid endemic countries, and in December 2017 the first typhoid conjugate vaccine was prequalified by WHO [10]. New data on the age-specific occurrence of typhoid fever from low- and middle-income countries show that typhoid fever tends to be common in the 0–4 years age group, with a large proportion of disease between 6 months and 2 years of age [9–11]. However, data on the occurrence of typhoid fever by age in Africa are limited.

Timely and appropriate antimicrobial therapy is needed to prevent complications and death from typhoid fever. However, antimicrobial resistance has been a growing problem in *Salmonella enterica*, compromising antimicrobial management. Traditionally, ampicilin, chloramphenicol, and trimethoprim-sulfamethoxazole were the drugs of choice for management of invasive *Salmonella* infections worldwide. However, the emergence of resistance to all of these agents, termed multidrug resistance (MDR) [9–11], lead to adoption of fluoroquinolones as drugs of choice. Fluoroquinolone-resistant *Salmonella* Typhi is now widespread in Asia [11, 12] and present in some parts of Africa [11, 12]. In 2017 the WHO identified fluoroquinolone-resistant *Salmonella enterica* among the priority pathogens for research and development of new antimicrobial agents [13]. More recently, extended spectrum beta lactamase (ESBL)-producing *Salmonella* Typhi has emerged and spread in South Asia [14]. Typhoid conjugate vaccines are the first to be approved on the basis of control of drug-resistant infections [5].

In order to inform typhoid conjugate vaccine policy in Tanzania and sSA, we sought to understand the role of *Salmonella* Typhi as a cause of community-acquired bloodstream infections in a rural setting in Tanzania, to examine patterns of antimicrobial resistance among isolates, and to describe the occurrence of typhoid fever by age.

## METHODS

### Study Site

**Figure 1.**
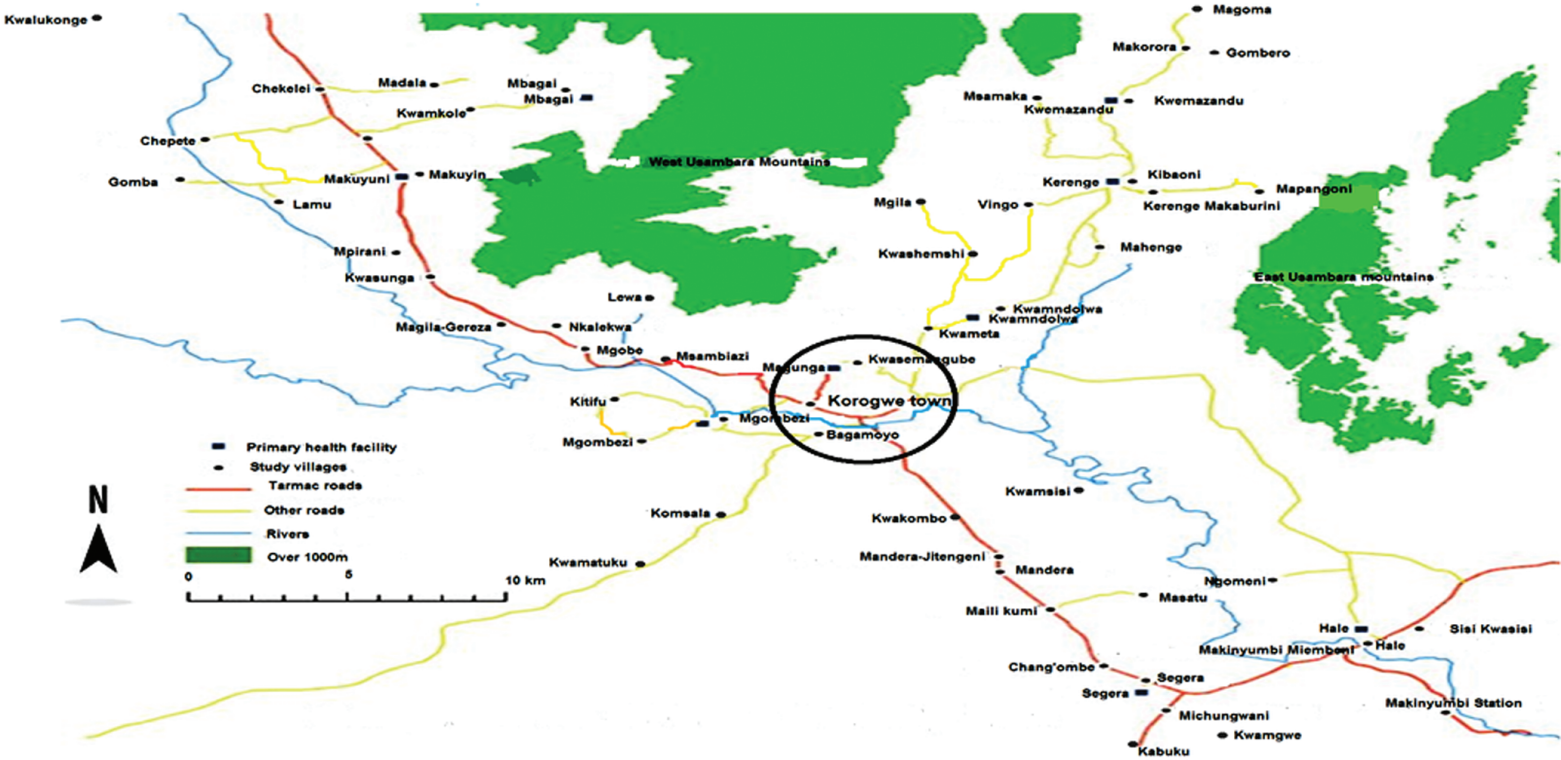
Map of Korogwe showing residential addresses of *Salmonella enterica* patients.

Korogwe District Hospital (KDH) is situated in Korogwe District in northeastern Tanzania (Figure 1). KDH with 145 inpatient and 28 pediatric beds serves as the main primary referral facility for approximately 261 000 people. The majority of communities surrounding Korogwe town use surface water from the Pangani River for drinking and other domestic use.

From August 2008 to April 2016, we operated a hospital-based surveillance system for the detection of severe malaria and other comorbidities among children admitted to KDH before and during implementation of the RTS, S/AS01E malaria vaccine trial [15]. RTS,S is a scientific name given to this malaria vaccine candidate and represents its composition. The ‘R’ stands for the central repeat region of *Plasmodium* (P.) falciparum circumsporozoite protein (CSP); the ‘T’ for the T-cell epitopes of the CSP; and the ‘S’ for hepatitis B surface antigen (HBsAg). These are combined in a single fusion protein (‘RTS’) and co-expressed in yeast cells with free HBsAg. The ‘RTS’ fusion protein and free ‘S’ protein spontaneously assemble in ‘RTS,S’ particles. Following the completion of RTS, S/AS01E malaria vaccine trial, the protocol was adopted as part of routine care for all febrile children admitted to the pediatric ward.

### Study Participants

During the study period, participants were identified prospectively among all admissions to the pediatric ward at KDH. All admitted patients aged 1 month to 5 years with an axillary temperature of ≥37.5° C at the time of admission or with a history of fever within 24 hours preceding admission were invited to participate. A standardized clinical history and physical examination was performed on consented participants by trained staff.

### Sample Collection and Laboratory Methods

Following skin cleaning and disinfection, 1–3 mL of venous blood was collected by venipuncture, inoculated into blood culture bottles, and incubated in an automated blood culture instrument (BACTEC 2050, Becton Dickinson, Franklin Lakes, NJ; BacTAlert, bioMérieux, Durham, NC). Broth from positive blood culture bottles was plated on MacConkey agar, Columbia agar enriched with 5% sheep blood, and chocolate agar (Oxoid, Hampshire, United Kingdom). The following organisms were classified as contaminants: coagulase- negative *Staphylococcus* spp., *Micrococcus* spp., *Propionibacterium* spp., coryneform bacteria, and *Bacillus* spp. and viridans streptococci. *Salmonella* strains were identified by API 20E biochemical testing (bioMérieux, Marcy L’Etoile, France) and confirmed by slide agglutination using antibody reagents specific for serogrouping Vi antibodies (Difco *Salmonella* Antiserum, Becton Dickinson, Franklin Lakes, NJ USA). Antimicrobial susceptibility testing was done by disc diffusion and interpreted according to British Standards for Antimicrobial Susceptibility testing interpretive criteria [16]. All isolates were tested for amoxicillin, ceftriaxone, chloramphenicol, and trimethoprim-sulfamethoxazole. Ciprofloxacin susceptibility testing was by E-test (Oxoid). Isolates were interpreted as ciprofloxacin susceptible with a minimum inhibitory concentration (MIC) ≤0.06 μg/mL, as intermediate (reduced susceptibility) with an MIC < 1 μg/mL and > 0.06 μg/mL and as resistant with an MIC ≥1 μg/mL [16]. Ceftriaxone was used as a screening drug for the detection of extended spectrum beta lactamase (ESBL)-producing strains. Resistance to amoxicillin, chloramphenicol, and trimethoprim-sulfamethoxazole defined MDR. All laboratory procedures were performed according to internationally acceptable standards and external quality assurance monitored by the Contract Laboratory Services (CLS) group, Pretoria, South Africa.

### Statistical Analysis

Data were double entered and validated using Microsoft Office access database 2007 (Microsoft Corporation, Redmond, WA), and statistical analyses were performed using Stata version 13 (StataCorp, Lake Way Drive, College Station, TX).Categorical data were displayed using frequencies and percentages, and continuous data were displayed using the median and interquartile range (IQR). Differences for parametric continuous variables among *Salmonella enterica* infected and uninfected patients were tested using analysis of variance for normally distribution or Mann-Whitney if for skewed parameters. A χ^2^ or Fisher exact test was used to compare categorical variables.

### Research Ethics

The Tanzania National Health Research Ethics Committee approved the protocol, reference number NIMR/HQ/R.a/Vol.IX/701a. Parents or caretakers of infants and children provided written informed consent.

## RESULTS

**Figure 2.**
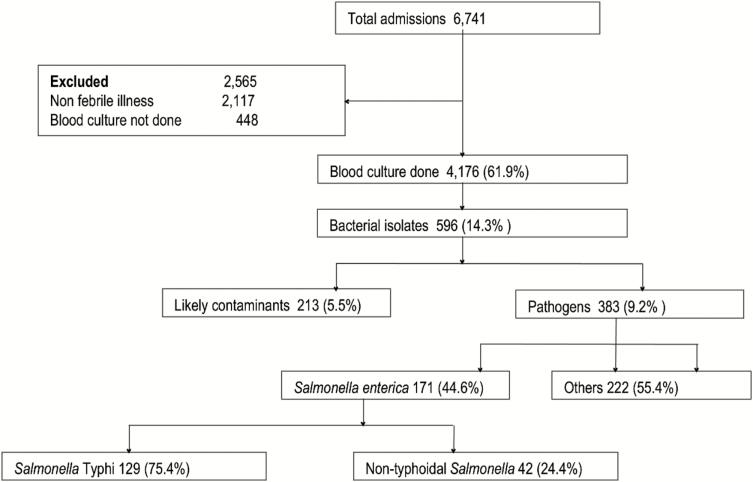
Flow diagram of febrile children with blood stream infections in Korogwe Tanzania, 2008–2016.

Of 6741 children admitted and screened for clinical features of febrile illness or history of fever, 3742 (55.5%) were males and 2999 (44.5%) were females. The blood cultures were collected from 4176 (61.9%) patients who met the inclusion criteria (Figure 2). The median (IQR) age was 14.8 (1.0–59.99) months. Of 4176 blood culture samples, 596 (14.3%) grew bacterial isolates. After exclusion of 213 likely contaminants, 383 (9.2 %) children yielded a pathogen of which 171 (44.6%) were *Salmonella enterica*. Of 171 children with blood cultures yielding *Salmonella enterica*, 129 (75.4%) were *Salmonella* Typhi, and 42 (24.6%) were nontyphoidal *Salmonella* (NTS). Of 107 participants with typhoid for whom their residence was known, 99 (92.5%) lived in rural areas (villages surrounding Korogwe town council). Pathogens other than *Salmonella enterica* included 75 (19.6%) *Streptococcus pneumoniae,* 7 (1.8%) *Streptococcus pyogenes,* 2 (0.5%) *Streptococcus agalactiae*, 16 (4.2%) other pathogenic *Streptococci*, 27 (7.0%) had *Escherichia coli,* and 15 (3.9%) had other bacterial pathogens (Figure 2). Of 4176 patients with blood cultures, parents or caretakers gave consent for human immunodeficiency virus testing in 2036 (48.8%) parents/caretakers who consented; of 66 (3.2%) children who were seropositive, only 2 had *S.* Typhi, and none had NTS infections.

**Figure 3.**
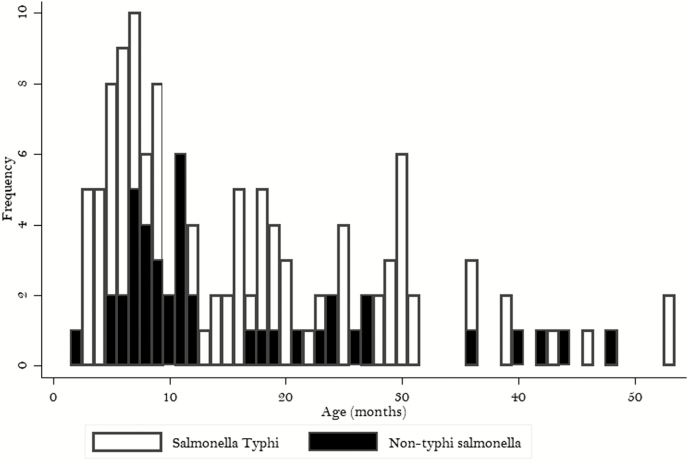
Age distribution of children with *Salmonella enterica* blood stream infections in Korogwe, Tanzania, 2008–2016.

Demographic and clinical characteristics of participants with *Salmonella* Typhi and NTS bloodstream infection are shown in Table 1. Figure 3 shows the occurrence of *Salmonella* Typhi and NTS bloodstream infection by age in months. The median (IQR) age of participants with *Salmonella* Typhi infection was 13.1 (2.4– 59.9) months, and for NTS it was 11.5 (2.0–48.0) months. Of 129 *Salmonella* Typhi bloodstream infections, 88 (68.2%) occurred <2 years of age and 68 (52.3%) at <1 year of age. Of 42 NTS bloodstream infections, 31 (73.8 %) occurred <2 years of age, and 24 (57.1%) occurred <1 year of age.

**Table 1.** Demographic and Clinical Characteristics of Children With *Salmonella enterica* Blood Stream Infections in Korogwe, Tanzania, 2008–2016

		*Salmonella* Typhi			Nontyphoidal *Salmonella*			
	All	YES	NO	*P* value	All	YES	NO	*P* value
Characteristics	N	n (%)	n (%)		N	n (%)		
Sex	4176	129	4025	.062	4176	42	4134	.753
Male		81 (62.8)	2205 (54.5)			26 (61.9)	2262 (54.7)	
Female		48 (37.2)	1842 (44.5)			16 (38.1)	1872 (45.3)	
Age (months), median (range)	4155	13.1 (2.4– 59.9)	15.1 (1.0–59.9)	.185	4155	11.5 (2.0–48.0)	15.0 (1.0–59.9)	.060
Axillary temperature (°C), mean ± SD	4156	38.3 ±1.0	38.0 ±1.1	.214	4156	38.3±1.1	38.1±1.0	0.480
Clinical presentation								
Fever	4156	129	4027	.076	4156	42		.353
Yes		102 (79.1)	2898 (72.0)			33 (78.6)	1147 (27.9)	
No		27 (20.9)	1129 (28.0)			9 (21.4)	2967 (72.1)	
Cough	4172	128	4044	.332		39	4133	.751
Yes		71 (55.5)	2067 (51.1)			19 (48.7)	2119 (51.3)	
No		57 (44.5)	1977 (48.9)			20 (51.3)	2014 (48.7)	
Diarrhea	4171	126	4045	.476	4171	40	4131	.699
Yes		44 (34.9)	1539 (38.1)			14 (35.0)	1569 (38.0)	
No		82 (65.1)	2506 (61.9)			26 (65.0)	2562 (62.0)	
Vomiting	4174	127	4047	.796		42	4132	.744
Yes		41 (32.3)	1351 (33.4)			15 (35.7)	1377 (33.3)	
No		86 (67.7)	2696 (66.6)			27 (64.3)	2755 (66.7)	
Concurrent illness								
Malaria^a^	4169	122	4047	.918	4169	42		.549
Yes		25 (20.5)	814 (20.1)			10 (23.8)	829 (20.1)	
No		97 (79.5)	3233 (79.9)			32 (76.2)	3298 (79.9)	
Upper respiratory tract infection	4172	127	4045	.609	4172	40		.893
Yes		62 (48.8)	1977 (48.9)			20 (50.0)	2110 (51.1)	
No		65 (51.2)	2068 (51.1)			20 (50.0)	2022 (48.9)	
Pneumonia^b^	4175	129	4046	.259	4175	31		.369
Yes		48 (35.7)	1349 (33.3)			11 (26.8)	1384 (33.5)	
No		83 (64.3)	2697 (66.7)			20 (73.2)	2750 (66.5)	
HIV infection^c^	2026	129	1897	.436		42	1984	.400
Yes		2 (1.5)	64 (3.4)		2026	0 (0.0)	66 (3.3)	
No		127 (98.5)	1833 (96.6)			42 (100.0)	1918 (96.7)	

Abbreviations: HIV, human immunodeficiency virus; SD, standard deviation.

^a^Malaria diagnosis was based on rapid test.

^b^Pneumonia was defined as presence of fever, cough, and difficulty in breathing.

^c^HIV test was performed only for children whom parents/caretakers provided consent after voluntary counseling.

Among 129 *Salmonella* Typhi tested, 89 (89.9%) were resistant to amoxicillin, 85 (81.0%) to chloramphenicol, and 93 (92.1%) to trimethoprim-sulfamethoxazole compared. Among 35 NTS tested, 22 (62.9%) were resistant to amoxicillin, 15 (39.4%) to chloramphenicol, and 27 (79.4%) to trimethoprim-sulfamethoxazole. Of *Salmonella* Typhi, 68 (81.0%) were multiple-drug resistant, and 6 (6.2%) were resistant to ciprofloxacin. Of NTS, 12 (41.4%) of NTS were multiple-drug resistant, and none was resistant to ciprofloxacin (Table 2). ESBL production was not detected in any of the *Salmonella enterica* isolate.

**Table 2.** Antimicrobial Susceptibility Pattern of *Salmonella* Typhi and Nontyphoidal *Salmonella* Bloodstream Isolates, Korogwe District Hospital, Korogwe, Tanzania, 2008–2016

	Proportion resistant			
Antimicrobial		*Salmonella* Typhi, n (%)	N	Nontyphoidal *Salmonella,* n (%)
Amoxicillin	99	89 (89.9)	35	22 (62.9)
Chloramphenicol	105	85 (81.0)	38	15 (39.4)
Trimethoprim-sulfamethoxazole	101	93 (92.1)	34	27 (79.4)
Ciprofloxacin	97	6 (6.2)	32	0 (0.0)
Ceftriaxone	97	0 (0.0)	32	0 (0.0)
Multidrug resistant^a^	84	68 (81.0)	29	12 (41.4)

^a^Resistant to amoxicillin, chloramphenicol, and trimethoprim-sulfamethoxazole.

## DISCUSSION

We found that *Salmonella* Typhi followed by NTS were the leading causes of community-acquired bloodstream infection among febrile infants and children admitted to Korogwe District Hospital in northeastern Tanzania. Furthermore, MDR was common among *Salmonella* Typhi. Furthermore, the majority of typhoid fever and NTS invasive disease occurred in infants <1 year of age.

Although others have suggested that typhoid fever in Africa might be concentrated in urban areas and informal settlements [6], our study supports a more recent work confirming that typhoid fever is common in rural parts of sSA. The finding that typhoid fever is common in rural areas in East Africa is perhaps not surprising given that many people still lack access to improved water and sanitation facilities [5]. It is likely that typhoid vaccination strategies targeting populations in urban areas and informal settlements would fail to prevent substantial amounts of typhoid fever occurring among the vast swathes of Africa whose population remains rural.

A systematic review of community-acquired bloodstream infections studies from Africa in 2010 showed that NTS predominated in sSA with only North African studies showing a predominance of *Salmonella* Typhi [2]. More recent work demonstrates the considerable variation of *S. enterica* bloodstream infections in both time [17] and place [7], with *Salmonella* Typhi being the leading cause of community-acquired bloodstream infections in some locations, including northeastern Tanzania [18]. We have previously reported that *Salmonella* Typhi and NTS bloodstream infections occurred at similar prevalence among febrile pediatric outpatients at KDH [19]. We now demonstrate that *Salmonella* Typhi is by far the most common cause of bloodstream infection among febrile pediatric inpatients. Since around 2005, there has been marked decline in malaria transmission intensity in most areas of sSA including northeastern Tanzania [20–23]. Of interest in northeastern Tanzania, the decline in malaria transmission has occurred at the same time as an increase in the role of *Salmonella* Typhi and a decline in the role of NTS as a cause of *Salmonella enterica* bloodstream infection [20, 24].

We found that more than half of *Salmonella* Typhi isolates were multiple-drug resistant and hence would not respond to the traditional first-line antimicrobials amoxicillin, chloramphenicol, and trimethoprim-sulfamethoxazole. Furthermore, a substantial minority of strains were also resistant to ciprofloxacin. Although the high proportion of multiple-drug resistant isolates is consistent with studies conducted elsewhere in sSA [25, 26], the occurrence of resistance to alternative agents such as ciprofloxacin is of great concern. Judicious use of antimicrobials is an imperative, but the specter of untreatable typhoid fever looms, and prevention by both nonvaccine and vaccine measures become increasingly important.

Our study had a number of limitations. First, the study was hospital-based study and therefore biased toward severely ill children and potentially missing milder cases. This could have underestimated the total number of invasive *Salmonella* infections in the community. Second, the hospital is a primary referral facility; many children may have received antimicrobial treatment prior to admission, which may have reduced the number of isolates seen in this study.

In conclusion, we demonstrate that *Salmonella* Typhi is the leading cause of community-acquired bloodstream infection in a rural district in northeastern Tanzania, that the majority of typhoid fever infections occurred in children aged <1 year, and that multiple-drug resistance was common. Our findings suggest that efforts to increase access to microbiologically safe water and food and improved sanitation be supported with routine use of typhoid conjugate vaccine early in infancy, including in rural areas of sSA.

## Notes

***Acknowledgments.*** The authors greatly acknowledge the children and parents who participated in this study, GlaxoSmithKline (GSK) and Partnership for Appropriate Technology in Health–Malaria vaccine initiative (PATH-MVI), which provided equipment and reagents as part of improving pediatric care in Korogwe District Hospital for conducting phase 3 and extension RTS, S/AS01E malaria vaccine trial. We would also like to acknowledge all research nurses and laboratory technicians at NIMR Korogwe. We also acknowledge the contributions from Contract Laboratory Services, South Africa, who provided external quality assurance services.

***Financial support.*** This work was supported by the Bill & Melinda Gates Foundation through PATH-MVI, through contract agreement number CTA4585_AMDI_MAL055 and the GSK Biologicals (funding number 110649).

***Supplement sponsorship.*** This supplement is sponsored by the Center for Vaccine Development and Global Health (CVD) at the University of Maryland School of Medicine.

***Potential conflicts of interest.*** All authors: No reported conflicts. All authors have submitted the ICMJE Form for Disclosure of Potential Conflicts of Interest. Conflicts that the editors consider relevant to the content of the manuscript have been disclosed.

Presented in part. 10th International Conference on Typhoid and Other Invasive Salmonellosis, Kampala, Uganda, 4–6 April 2017, abstract Invasive *Salmonella* Typhi in Korogwe Tanzania
